# Distributed Wireless Neural Recording System for Multi-Region Brain Activity Monitoring

**DOI:** 10.3390/bios16070370

**Published:** 2026-07-07

**Authors:** Liu Yang, Changhua You, Gang Wang, Xuan Zhang, Canyang Wang, Bo Cheng, Zhengtuo Zhao, Ning Xue, Lei Yao

**Affiliations:** 1 School of Integrated Circuits, Shanghai Jiao Tong University, Shanghai 201100, China; yangliu0606@sjtu.edu.cn; 2 Lingang Laboratory, Shanghai 201100, China; 3 State Key Laboratory of Transducer Technology, Aerospace Information Research Institute (AIR), Chinese Academy of Sciences, Beijing 100190, China; youchanghua18@mails.ucas.ac.cn; 4 Institute of Flexible Electronics Technology of Tsinghua, Jiaxing 314000, China; wanggang@ifet-tsinghua.org; 5 School of Aerospace, Tsinghua University, Beijing 100084, China; 6 Mtrix Technology Co., Ltd., Shanghai 201800, China; zhangxuan@mtrix.cn; 7 StairMed Technology Co., Ltd., Shanghai 200131, China; acan_vet@163.com (C.W.); cb_ymj@163.com (B.C.); 8 State Key Laboratory of Neuroscience, Institute of Neuroscience, CAS Center for Excellence in Brain Science and Intelligence Technology, Chinese Academy of Sciences, Shanghai 200031, China; zhaozt@ion.ac.cn

**Keywords:** wireless neural recording, multi-region brain monitoring, neural recording ASIC, parallel-link architecture, neural signal detection, compression

## Abstract

Distributed neural interfaces for multi-region implantation require both scalable interconnects and robust telemetry, yet conventional centralized or fully distributed architectures often trade-off wiring complexity, resource reuse, and transmission stability. This work presents a distributed wireless neural recording system based on a parallel-link architecture and a custom 12-channel neural recording Application-Specific Integrated Circuit (ASIC). Each remote module is connected to a central hub through an independent four-wire link (VDD/GND/LVDS±). The ASIC integrates modular digital pixels (MDPs), an on-chip oscillator, a Manchester encoding, and a Low-Voltage Differential Signaling (LVDS) output to reduce interconnect count while maintaining reliable serial transmission. Fabricated in SMIC 0.18 μm CMOS, the chip occupies 4.84 mm × 0.36 mm and consumes 10.13 mW in total, with 48.5 μW/channel consumed by the recording channels excluding the LVDS driver. It achieves 5.6 μVrms input-referred noise and a measured per-channel sampling rate of 28.93 kSps. A compact 20 mm^2^ recording module and an FPGA-based central hub with real-time decoding and compression were implemented for validation. In vivo mouse experiments demonstrate clear action-potential recordings across 12 channels, confirming the feasibility of stable and scalable multi-region neural signal acquisition.

## 1. Introduction

The human brain contains approximately 100 billion neurons connected through extensive synaptic networks that form specialized functional circuits [1]. Motor and sensory functions are driven by coordinated neural activity across multiple regions, such as the cortex, thalamus, and spinal cord, rather than by isolated neurons. Therefore, simultaneous recording from spatially distributed brain regions is essential for understanding cross-scale brain dynamics and the neural basis of motion and cognition in humans and primates [2,3]. Recent studies, for example, have jointly analyzed activity in the primary motor cortex (M1), dorsal premotor cortex (PMd), ventral premotor cortex (PMv), and prefrontal cortex (PFC) to reveal neural control rules in natural behavior [4,5]. These needs create a strong demand for multi-channel systems that can monitor multiple brain regions in freely moving subjects. However, achieving flexible multi-region implantation while keeping implantable hardware compact and lightweight remains a major challenge.

**Figure 1:**
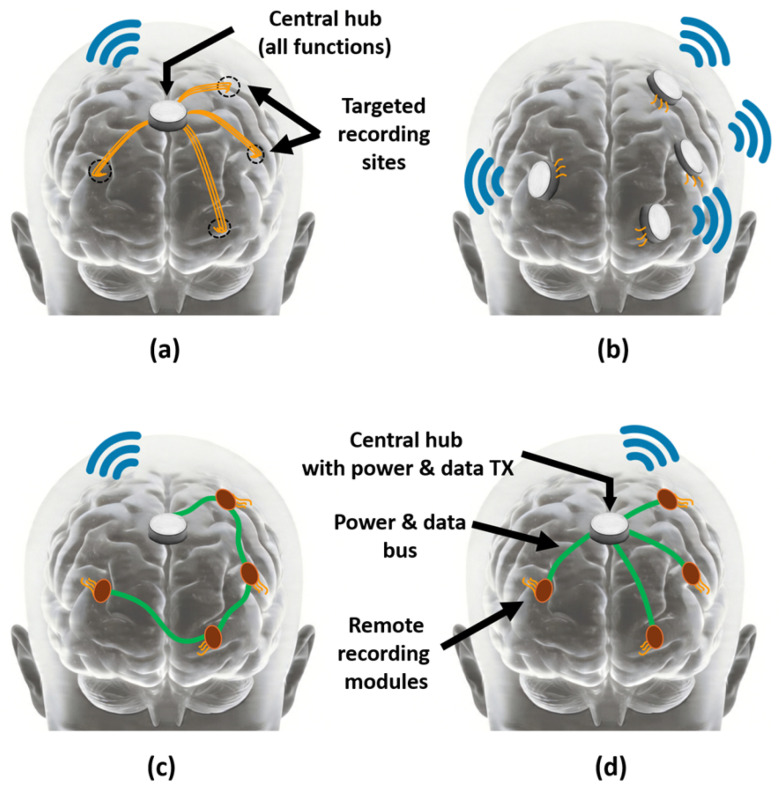
Comparison of implantable neural recording system architectures for multi-region brain monitoring: (**a**) Centralized architecture: all electrodes are routed to a single hub that houses the shared processing, telemetry, and power modules. Wiring complexity grows rapidly with the number of recording sites; (**b**) Fully distributed architecture: each recording region carries an independent local hub with its own wireless link and power receiver, eliminating long interconnect bundles at the cost of per-module wireless power delivery and cross-region synchronization; (**c**) Hybrid distributed architecture with serial bus: front-end processing is placed near the recording sites while a shared serial bus connects remote modules to a centralized backend for power and data aggregation. Scalability is constrained by cascading-failure risk, IR drop, and increasing bus data-rate pressure; (**d**) Proposed parallel-link architecture: each remote recording module connects to the central hub through an independent four-wire link (VDD/GND/LVDS±), enabling independent operation and straightforward scaling without bus contention or single-point-of-failure propagation.

A typical implantable wireless neural recording system can be described by four functional blocks: neural electrodes, local neural signal processing, data telemetry, and power management. The electrodes transduce extracellular neural activity into electrical signals, and the neural signal processing block performs amplification, filtering, and analog-to-digital conversion. The telemetry block transmits processed data through a wireless protocol, while power management harvests, regulates, and distributes energy across the system. Conventionally, the most straightforward architecture places electrodes in different brain regions and routes them back to a central hub that contains the shared processing, telemetry, and power modules, as shown in Figure 1a [6,7,8]. This architecture is efficient in resource sharing, but wiring complexity and signal integrity become major bottlenecks as the number of electrodes and recording regions increases. To address this issue, several distributed neural recording architectures have been reported [9,10,11,12,13,14,15,16]. In [9,10,11,12,13,14], each recording region includes an independent local hub, which eliminates large interconnect bundles but introduces challenges in multi-region wireless power delivery, wireless networking, and cross-region timing alignment, as shown in Figure 1b. In [15,16], a hybrid architecture distributes front-end signal processing modules near the recording sites and connects them through a shared serial bus to a centralized backend for shared power management and wireless telemetry, as shown in Figure 1c. This approach is attractive because it shortens sensitive analog interconnects while preserving efficient shared backend resources. However, when remote modules are connected serially through shared power/data wires, scalability can be constrained by design challenges such as cascading-failure risk, power-integrity degradation (for example, IR drop), inter-module interference, and increased high-speed wireline bandwidth demand as channel count and region count grow.

To address the problems above, we propose a distributed neural recording system with a parallel-link architecture, as shown in Figure 1d. Each remote recording module is connected to the central hub through an independent four-wire supply-and-data link, allowing multiple modules to operate in parallel and thereby alleviating scalability limitations in [16]. The central hub performs shared power management and data processing/transmission. Each remote module consists of a 12-channel flexible neural probe and a 12-channel recording Application-Specific Integrated Circuit (ASIC). To improve signal integrity while keeping link pin count low, the recording ASIC integrates an on-chip clock source, a Manchester encoder, and a Low-Voltage Differential Signaling (LVDS) output circuit. The central hub is implemented with an FPGA and a Bluetooth module for data acquisition, processing, and wireless transmission. The proposed system is validated through in vivo mouse experiments, demonstrating effective capture of high-quality neural signals.

This paper is organized as follows: Section 2 describes the system and module architecture, Section 3 presents the design of the core 12-channel neural recording ASIC, Section 4 reports bench-top and in vivo experimental results, and Section 5 concludes the paper.

## 2. System Overview

**Figure 2:**
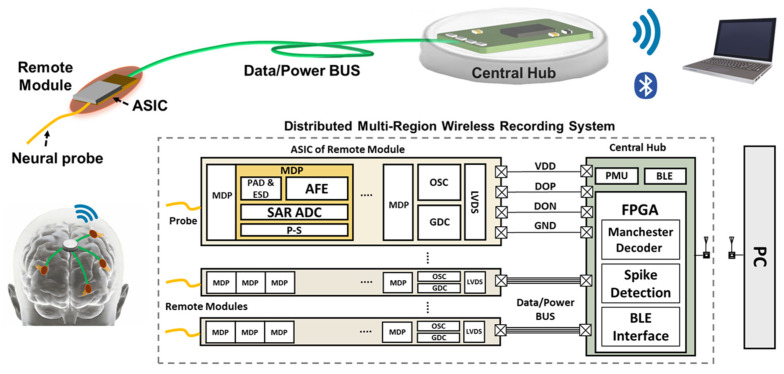
Distributed multi-region neural recording system architecture. Each remote module connects to the central hub through an independent four-wire link (VDD/GND/LVDS±) carrying both power and Manchester-coded serial data. The central hub integrates power management, FPGA-based signal processing (Manchester decoding, spike detection, data compression), and Bluetooth transmission for real-time multi-region neural signal recording.

As illustrated in Figure 2, the proposed distributed neural recording system consists of three major parts: remote modules, central hub, and data/power buses in-between. Each remote module houses a 12-channel flexible neural probe and a 12-channel custom-designed ASIC, acquiring and conditioning the wide-band neural signals. The central hub processes the data from remote modules and transmits the data to an external station through Bluetooth. The power is provided to remote modules through a 2-wire power line and data is transmitted from remote modules to the central hub through a 2-wire LVDS data line. This parallel-link topology eliminates the cascading-failure and power-integrity risks of serial-bus architectures [16] while maintaining compact wiring for multi-region neural recording.

### 2.1. Remote Recording Module Overview

The remote recording module comprises a 12-channel neural recording ASIC wire-bonded to a flexible neural probe. The ASIC integrates 12 Modular Digital Pixels (MDPs) [17], an on-chip ring oscillator (OSC), a Global Digital Controller (GDC), and an LVDS driver.

Each MDP contains a complete signal processing chain: an Analog Front-End (AFE) for amplification and filtering, a 12-bit successive approximation register (SAR) ADC for digitization, a Parallel-to-Serial (P-S) converter, and pad/ESD circuitry for probe connection. During operation, extracellular neural signals sensed by the probe are amplified and filtered by the AFE, digitized by the SAR ADC, serialized by the P-S converter, and forwarded to the GDC. The GDC aggregates data from all 12 MDPs into a single serial stream, which is Manchester-encoded to embed clock information and then driven off-chip through the LVDS output pair (DOP/DON). Manchester encoding eliminates the need for a dedicated clock wire, while LVDS provides high noise immunity for reliable data transmission over the extended site-to-hub interconnect.

### 2.2. Central Hub Overview

The central hub comprises a Power Management Unit (PMU), an FPGA, a Bluetooth Low-Energy (BLE) module and a battery. The PMU manages the power from the battery and supplies regulated power to all connected remote modules through independent VDD/GND lines. Because each module has its own power path, IR-drop accumulation and ground-bounce coupling between modules are avoided. The FPGA receives the Manchester-encoded LVDS data from each remote module, decodes it, and performs real-time neural spike detection and data compression. Due to the limited throughput of BLE, a spike detection algorithm and two customized data transmission modes are implemented in the FPGA to compress the neural data before wireless transmission. The detailed algorithm and frame formats are described in Section 3. The BLE module transmits the processed data wirelessly to an external receiver (laptop or mobile device), enabling real-time monitoring of neural activity in freely behaving subjects.

## 3. Circuit and System Implementation

### 3.1. Remote Recording Module Implementation

The remote recording module is designed to be compact and power-efficient while maintaining high signal integrity for neural recording. The core component is the custom-designed 12-channel neural recording ASIC, which integrates the entire signal processing chain for each channel, as well as shared digital control and data output circuitry. The design of the ASIC focuses on achieving low input-referred noise and robust data transmission capabilities while minimizing power consumption and chip area.

#### 3.1.1. AFE

**Figure 3:**
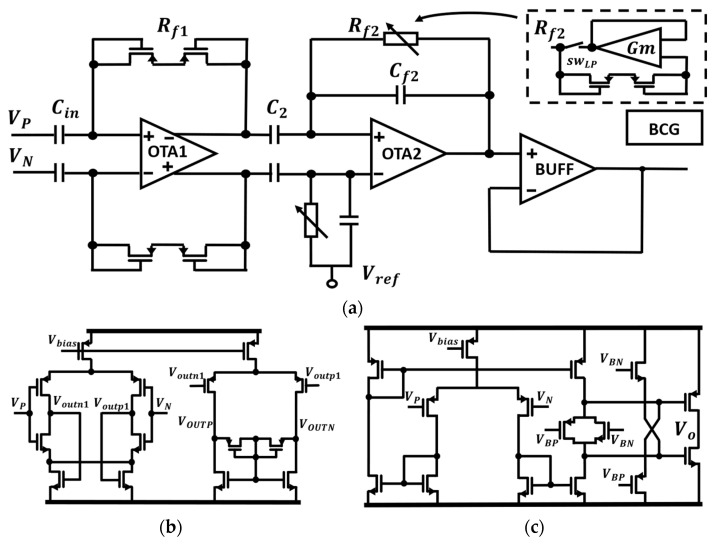
Schematic of: (**a**) AFE; (**b**) OTA in LNA; (**c**) two-stage OTA in BUFF.

The AFE consists of four main modules: (a) a low-noise amplifier (LNA), (b) a band-pass filter (BPF), (c) a buffer (BUFF), and (d) a bias current generator (BCG), as shown in Figure 3a. As the very first stage for processing small neural signals at ~μV level, the LNA is a critical component that dictates the noise performance and signal integrity of the entire recording chain. A capacitively coupled topology with natural DC isolation is used to eliminate electrode offset from the probe. Figure 3b shows the schematic of the Operational Transconductance Amplifier (OTA) in the LNA, which adopts a fully differential current-reuse structure. The input transistors are biased in the weak inversion region to maximize current efficiency [18,19]. Notably, to achieve high gain and improved noise performance within a minimal chip area, this design omits explicit feedback capacitors and instead utilizes the intrinsic parasitic capacitance to function as the feedback capacitor. Although parasitic capacitance values are subject to process variation, comprehensive PVT corner and Monte Carlo post-layout simulations confirm that the LNA gain remains approximately 56 dB with sufficient output headroom to avoid saturation even under worst-case conditions. The measured channel-to-channel gain uniformity (±1.8%, Section 4) further validates the viability of this approach.

In neural recording, two frequency bands are generally of interest: Local Field Potentials (LFPs) in the 0.5 Hz–300 Hz range, which reflect the average activity of neuronal populations, and Action Potentials (APs) in the 300 Hz–10 kHz range, representing individual neuronal firing responses. The proposed BPF supports both broadband and spike-detection modes by adjusting the feedback resistor R_f2_ to select between high-pass cutoff frequencies of 0.5 Hz and 300 Hz. It also functions as an anti-aliasing filter with a 3 dB low-pass frequency of 10 kHz, resulting in an overall AFE bandwidth of selectable 0.5/300 Hz–10 kHz. Furthermore, the BPF boosts the total gain to 62 dB to match the dynamic range of the ADC.

The BPF is followed by a unity-gain buffer using a two-stage OTA [20] (as shown in Figure 3c) to drive the ADC. The output stage uses a floating voltage source based on a trans-linear loop to achieve Class-AB operation. Since the charging or discharging current to the load is not limited by the quiescent current, this topology achieves a high slew rate while maintaining low quiescent current.

#### 3.1.2. SAR ADC

**Figure 4:**
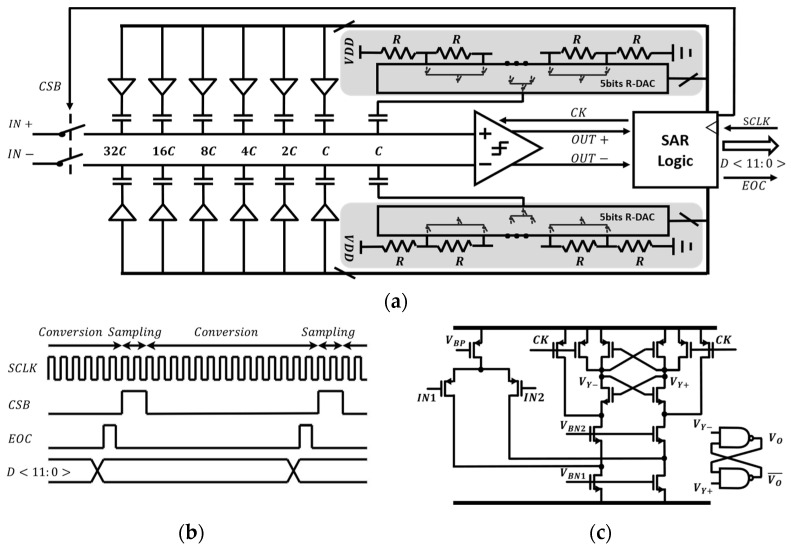
(**a**) Block diagram of SAR ADC with capacitor–resistor hybrid DAC; (**b**) timing diagram for operation of SAR ADC; (**c**) schematic of comparator.

A 12-bit SAR ADC is implemented to digitize the pre-processed neural signals from the AFE. The SAR ADC mainly consists of a DAC array, a dynamic comparator, and SAR digital logic circuits, as shown in Figure 4a. The operation timing diagram is shown in Figure 4b. The SAR ADC is designed for a nominal 500 kHz clock signal SCLK, and the signal CSB marks the start of A/D conversion. It takes 2 clock cycles to sample the output of the AFE and 14 clock cycles to complete the comparison and SAR operation, yielding a designed sampling rate of 31.25 kSps. After each conversion period, an End-of-Conversion (EOC) signal is generated, and the results are provided in parallel as D<11:0>.

To reduce the DAC area, a hybrid capacitor–resistor architecture is adopted, consisting of a 6-bit MSB capacitor array and a 5-bit LSB resistor array [21]. Notably, the use of top-plate sampling allows the MSB to be determined directly in the first comparison cycle, thus further saving one bit compared with conventional 12-bit SAR ADC structures. In terms of design parameters, the total resistance of the R-network is 1.28 MΩ (40 kΩ per unit), and the total capacitance of the C-network is 3.184 pF (49.75 fF per unit), with a unit area of 5 μm × 5 μm. To lower power consumption, the comparator uses a StrongARM latch topology [22] and integrates a folded-cascode structure to reduce kickback noise, as shown in Figure 4c.

**Figure 5:**
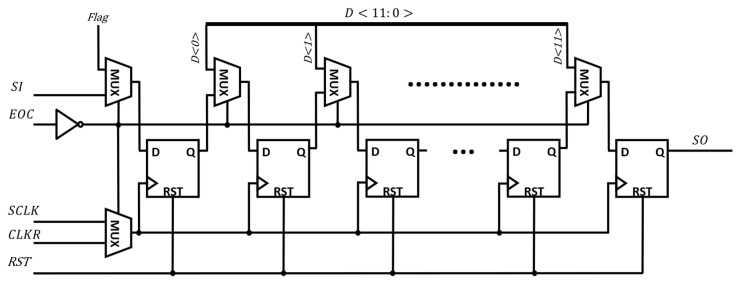
Parallel-to-Serial output unit of MDP.

To facilitate module connection and future expansion, a 13-bit P-S unit is used to store the conversion results. As shown in Figure 5, the multi-channel parallel ADC data is formatted into a single-wire serial output through the Serial Input (SI) and Serial Output (SO) signals.

#### 3.1.3. Global Digital Control

The GDC manages the timing and control of the entire MDP, including the ADC conversion process, data latching, and serialization. It generates control signals such as CSB and SCLK to coordinate the operation of the ADC and P-S unit. The GDC also interfaces with the on-chip OSC to synchronize the timing of data acquisition and transmission. The control logic operates as follows: First, after power-on, the RST signal resets all outputs of the D-flip-flops (DFFs). Then, at the end of a conversion, the ADC data is latched into the DFFs by the rising edge of SCLK during the high level of the EOC signal. Additionally, a CHK input is connected to the EOC output of the previous module to serve as a data parity bit for verification. Finally, based on the 16 MHz read clock signal CLKR, the data from all 12 channels are transferred to the output of the P-S unit during the low level of EOC. To reduce the number of external wires and eliminate the need for a dedicated clock line, Manchester encoding is applied to embed clock information in the serialized data stream before LVDS transmission.

#### 3.1.4. OSC

**Figure 6:**
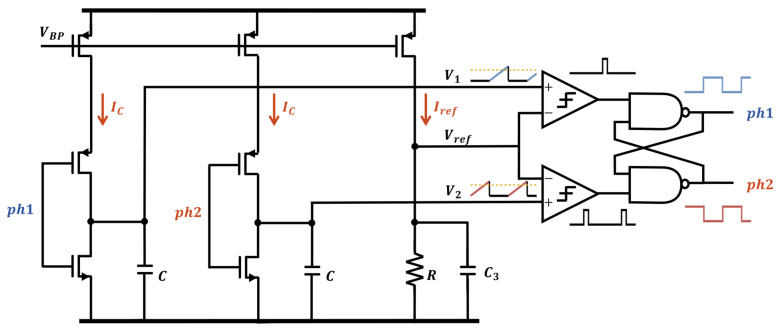
Schematic of RC relaxation oscillator.

To minimize external interference and reduce the number of interconnection wires, a conventional RC relaxation OSC [23] is implemented as the on-chip reference clock for the ADC and digital logic, as shown in Figure 6. The control circuit, consisting of two comparators and a latch, alternately connects each timing capacitor (C) to a bias-current source (I_C_) for charging or to the ground for discharging. The reference voltage *V_ref_* of the comparators is generated by a reference current *I_ref_* flowing through a reference resistor (R). In this structure, one clock period is equal to the sum of the RC delay and the loop delay of the comparators and logic circuits. Furthermore, first-order temperature compensation for the RC delay is achieved by connecting a Proportional-to-Absolute-Temperature (PTAT) resistor and a Complementary-to-Absolute-Temperature (CTAT) resistor in series. Post-layout simulations at the TT corner yield a nominal OSC frequency of approximately 16 MHz; the measured frequency on the fabricated chip is 14.81 MHz (Section 4), corresponding to a 7.4% deviation attributed to process variation.

#### 3.1.5. LVDS

**Figure 7:**
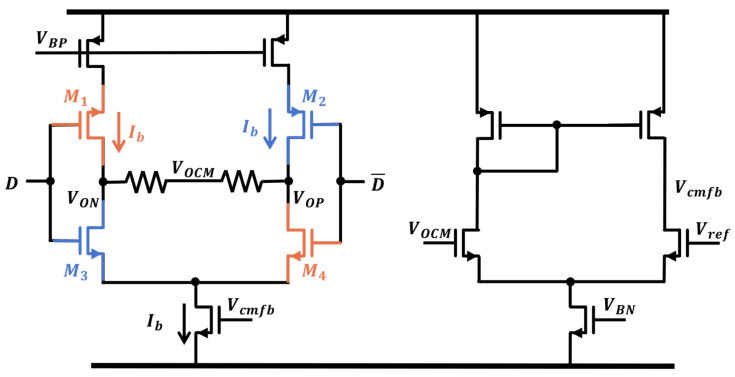
Schematic of LVDS driver.

To transmit the Manchester-encoded serial data off-chip with high noise immunity and extended range, an LVDS driver is employed. The driver adopts a bridge-switched topology [24], as shown in Figure 7. Four MOS switches (M_1_–M_4_) are arranged in an H-bridge configuration. When M_1_ and M_4_ are turned on, the output current flows in the forward direction, producing a positive differential voltage across the termination resistor. When M_2_ and M_3_ are turned on instead, the current direction reverses, inverting the differential output. This complementary switching scheme provides a constant-current output that minimizes supply noise coupling.

### 3.2. Central Hub Implementation

There are three main components in the central hub: the PMU, the FPGA, and the BLE module. The PMU regulates power from the battery and supplies it to the remote modules. The FPGA serves as the central processor, responsible for decoding the Manchester-coded neural data received from each remote module via the LVDS interface. A spike detection and data compression algorithm is implemented on the FPGA, which interfaces with the BLE module for real-time multi-channel spike-time transmission within the limited Bluetooth bandwidth. The BLE module enables wireless data transmission from the central hub to an external receiver, such as a laptop or mobile device, allowing for real-time monitoring of neural activity.

#### 3.2.1. PMU and BLE Hardware

The PMU generates multiple voltage domains through low-dropout regulators, including 3.3 V and 1.8 V supply rails. These supply rails power the FPGA, BLE module, digital interfaces, and remote acquisition modules. By separating the power domains, the proposed design improves supply stability and reduces the influence of digital switching noise on the weak neural signal acquisition path.

The BLE module is used for wireless data transmission between the central hub and an external receiver. In this system, an FSC-BT630 Bluetooth module is employed as the low-power wireless communication unit. The module is powered by the 3.3 V supply rail and communicates with the FPGA through UART_TX and UART_RX signals. The FPGA transfers the compressed spike information to the BLE module through the UART interface, and the BLE module subsequently transmits the data wirelessly to an external device, such as a mobile terminal. Considering the limited bandwidth of Bluetooth communication, the system does not directly transmit complete raw neural waveforms. Instead, it prioritizes the transmission of key spike-event information extracted by FPGA-based processing, thereby reducing the wireless data rate while enabling real-time monitoring of neural activity. This wireless transmission architecture reduces the constraints imposed by wired connections on animal movement and provides a hardware basis for in vivo neural recording under freely moving conditions.

#### 3.2.2. Detection and Compression Algorithm on FPGA

**Figure 8:**
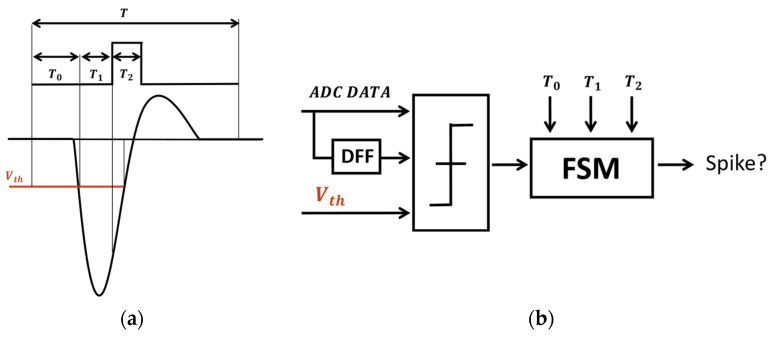
(**a**) Principle of spike detection algorithm; (**b**) block diagram of the FPGA-based spike detection algorithm.

The neural spike detection algorithm is based on the standard waveform of neuronal firing and adopts a dual-threshold detection principle [25,26], as shown in Figure 8. This detection is implemented using comparators and a Finite State Machine (FSM) within a predefined time window. By comparing the input signal with the thresholds and using the FSM to track the timing, the algorithm can accurately identify signal patterns that drop below the threshold and subsequently rise above it within a specific time range. This approach ensures reliable spike-event detection based on predefined criteria while minimizing false positives caused by noise.

**Figure 9:**
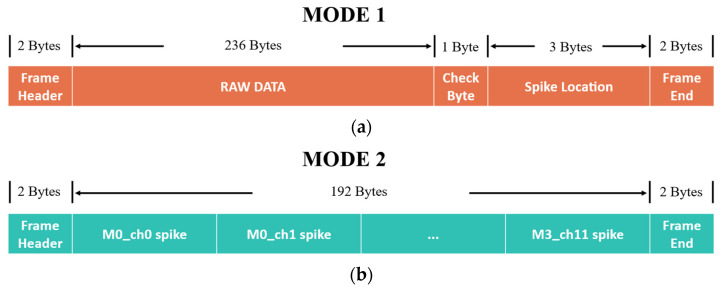
BLE data frame format for transmission: (**a**) mode 1; (**b**) mode 2.

Regarding data transmission, a single-channel ADC with the measured 28.93 kSps sampling rate and 12-bit resolution generates a data rate of approximately 347 kbps. Due to the throughput limitations of BLE, only a maximum of two channels of raw data can be transmitted in real time without compression. To overcome this bottleneck, two customized BLE data frame formats are proposed as shown in Figure 9:

Mode 1 (Hybrid Transmission Mode): Each data frame consists of a 2-byte frame header, 236 bytes of raw data (118 sampling points), a 1-byte check byte, 3 bytes of spike location information, and a 2-byte frame end. At the measured sampling rate of 28.93 kSps, a standard neural spike spans approximately 58 samples; therefore, 118 raw samples are sufficient to capture approximately two complete spike events per frame.

Mode 2 (All-Channel Spike Transmission Mode): To enable synchronized monitoring across all channels, Mode 2 transmits only extracted spike information. The frame structure includes a 2-byte frame header, a 192-byte spike data payload, and a 2-byte frame end. The 192-byte payload carries spike information from all 48-channel across four modules (M0–M3), achieving a data compression ratio of 98.7%. This mode enables real-time synchronized monitoring of all 48-channel over a single BLE link.

## 4. Measurement Results

**Figure 10:**
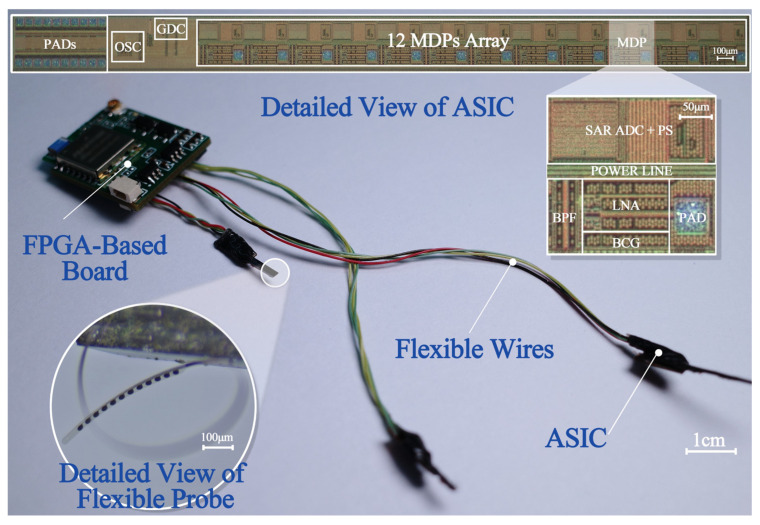
The wireless neural signal acquisition system.

The proposed custom-designed 12-channel neural recording ASIC was fabricated in the SMIC 180 nm standard CMOS process. Based on this ASIC, a customized lightweight 12-channel remote recording module was developed to establish a wireless system for distributed signal acquisition across multiple brain regions, as shown in Figure 10. The chip occupies a silicon area of 4.84 mm × 0.36 mm, including I/O pads.

**Figure 11:**
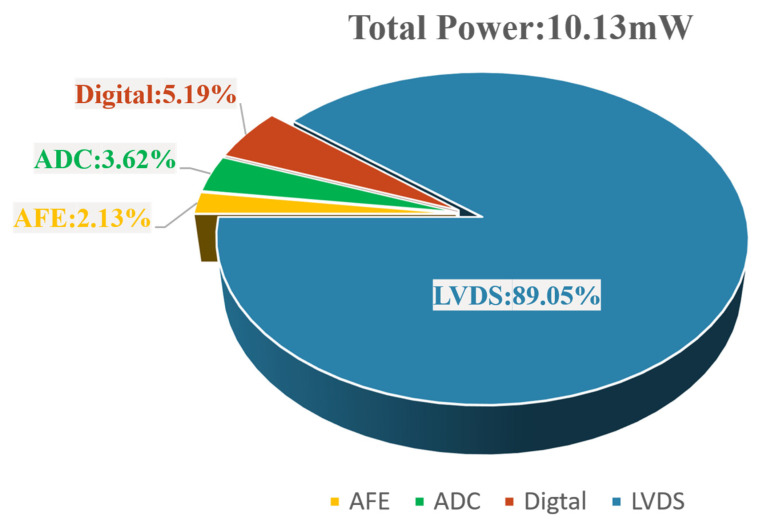
Power breakdown of the proposed neural-recording chip.

Measurements show that under a 1.8 V supply voltage, the total power consumption of ASIC is approximately 10.13 mW. A significant portion of this power is consumed by the LVDS driver, which draws approximately 5.3 mA. Although this represents a substantial power overhead for a neural recording chip, the LVDS interface provides high data bandwidth, robust noise immunity, and exceptional stability. These characteristics are indispensable for ultra-high-throughput neural acquisition, where real-time performance and signal integrity are paramount. The detailed power breakdown is illustrated in Figure 11.

### 4.1. ASIC Characterization

**Figure 12:**
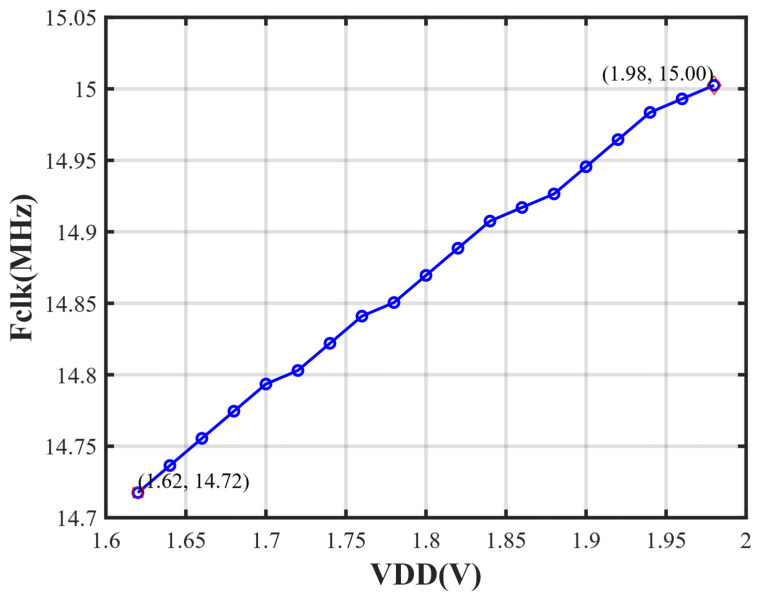
The relationship between OSC frequency and supply voltage.

Bench tests were conducted to verify the performance of the ASIC and the overall system. At a nominal voltage of 1.8 V, the on-chip OSC circuit produced an output clock frequency of 14.81 MHz. To evaluate the system’s robustness against power fluctuations, the supply voltage was varied by ±10% (from 1.62 V to 1.98 V), with the corresponding frequency variations recorded in Figure 12. The measured clock sensitivity was 0.78 MHz/V, corresponding to less than 10% frequency variation across this supply range.

With clocks derived from the OSC output, the actual ADC operation clock frequency is 462.88 kHz, and the divide-by-512 sampling clock gives each MDP a sampling rate of 28.93 kSps. Based on the system parameters, assuming each channel generates 13 bits of data per conversion, the 14.81 MHz read clock can theoretically support up to 39 channels. For designs requiring higher channel density, this can be achieved by either reducing the ADC sampling rate or increasing the read clock frequency.

**Figure 13:**
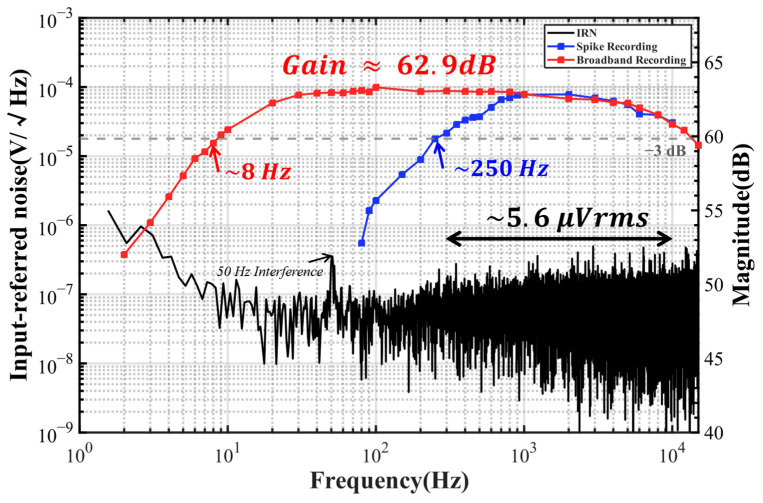
Measurement results of frequency response and input-referred noise.

**Figure 14:**
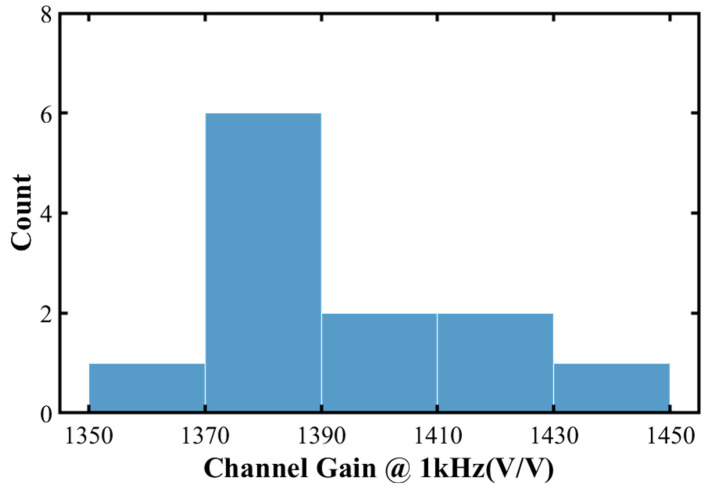
Measured gain variation across all channels in the in vitro setup.

**Figure 15:**
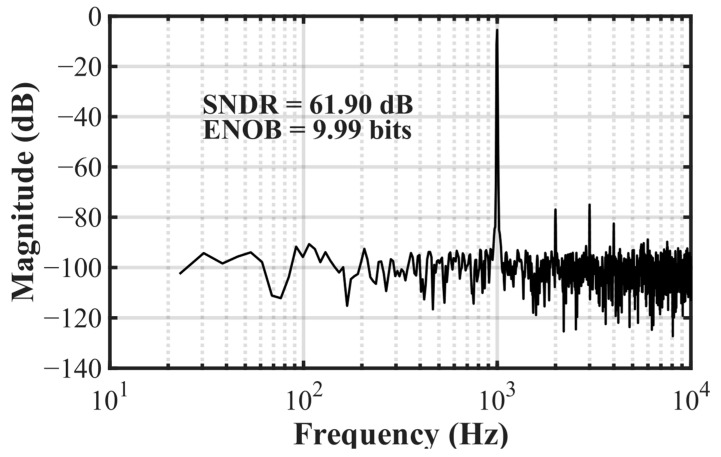
Measurement results of ENOB for the entire system under a 1 kHz input signal.

Figure 13 presents the measured transfer function and input-referred noise (IRN) of the entire recording signal chain (AFE + ADC). With LP set to 1 and 0, the measured mid-band gain is 62.9 dB, and the high-pass cutoff frequencies are 8 Hz and 250 Hz, respectively. Within the signal bandwidth of 250 Hz to 10 kHz, the measured IRN is 5.6 μVrms. Furthermore, Figure 14 shows the histogram of mid-band gain variations across the 12 channels. The results indicate an average voltage gain of 1392.5 *v*/*v*, with a deviation range strictly controlled within ±24.87 *v*/*v* (±1.8%). This consistency supports the feedback strategy described in the AFE subsection of Section 3 and indicates that utilizing parasitic capacitance as Cf under controlled layout is practical. These experiments indicate that this approach reduces chip area and enhances gain while maintaining excellent channel uniformity and system stability, supporting large-scale distributed neural signal acquisition. Figure 15 shows the measured signal-to-noise and distortion ratio (SNDR) of the ADC with a 1 kHz sine wave input. The ADC achieves an SNDR of 61.9 dB, which corresponds to an effective number of bits (ENOB) of 9.99 bits. This reported ENOB accounts for the noise sources of the entire system, including the thermal and flicker noise of the AFE, as well as the quantization noise and non-idealities of the ADC.

### 4.2. In Vivo Demonstration

**Figure 16:**
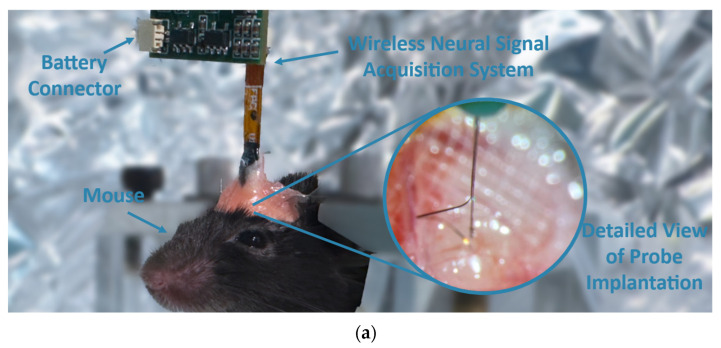

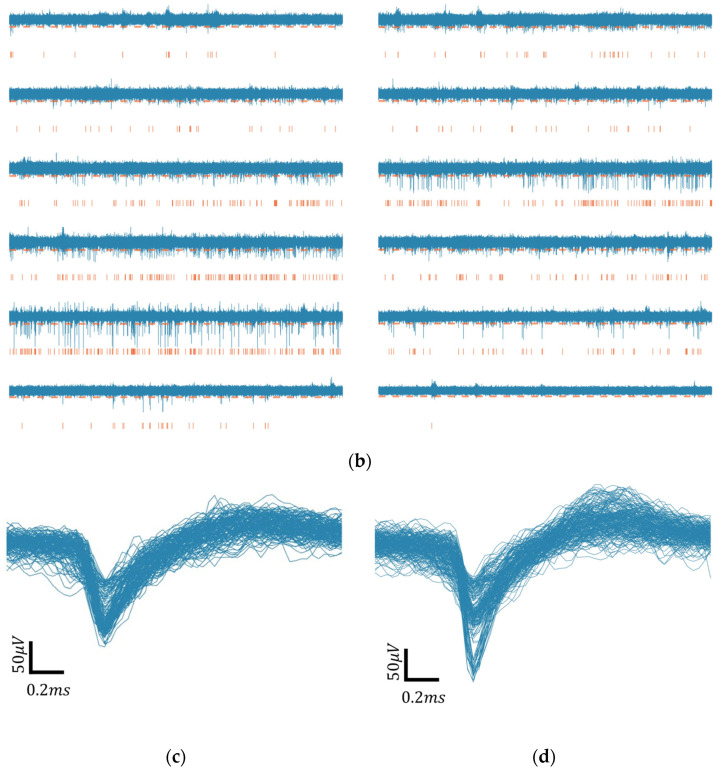
(**a**) In vivo experiment setup; (**b**) 30 s traces from 12 channels; scale bars correspond to 100 μV vertically and 2.5 s horizontally. The red vertical lines indicate detected spike events, and the red horizontal dashed lines indicate the thresholds used for spike detection; (**c**) overlaid spike waveforms from channel 5; (**d**) overlaid spike waveforms from channel 8.

The proposed neural signal acquisition module is formed by wire-bonding a custom flexible neural probe to the ASIC, which is then mounted on a flexible PCB with FPC interfaces. The 12-channel probe was fabricated following the process reported in [27]. The material of the probeconsists of Au, PDMS, and PEG. Au forms the electrodes and metal traces, PEG was used to temporarily increase the stiffness of the probe before implantation and dissolved after contact with brain tissue, while PDMS served as the exposed flexible insulation material. This integrated module, measuring 8 mm × 2.5 mm, can be used for multi-region brain implantation. To further validate its functionality and performance, a complete wireless neural signal recording system was developed for in vivo experiments, as shown in Figure 16a.

In the in vivo experiments, three adult male mice (C57BL/6, 8 weeks old) were used, and one 12-channel flexible probe was implanted in each mouse. The implantation surgery was performed under general anesthesia. The mice were anesthetized with 1.5–2% isoflurane and fixed on a stereotaxic apparatus, while body temperature was maintained at 37 °C to keep physiological conditions stable. Under aseptic conditions, the skull was exposed and a craniotomy was performed. The flexible probe in the neural signal acquisition module was then slowly implanted into the caudate putamen (CPu) using the stereotaxic coordinates ML ± 1.25 mm, AP 0.98 mm, and DV −4 mm. Ground electrode was used in the neural recording system. For the ground connection, a small hole was drilled through the skull without damaging the dura or brain tissue, and a bent silver wire was inserted into the hole to contact the skull and brain surface. Silicone was then used to seal the ground-electrode hole and the craniotomy region to protect the brain tissue. The probe and ground electrode were further stabilized using Super-Bond C&B adhesive and dental cement. After 5 days of postoperative care and observation, neural signal acquisition was performed in the awake and freely moving state. Four repeated recording experiments were conducted over a period of one month to preliminarily evaluate the repeated in vivo recording capability of the system.

All experimental procedures were conducted in strict accordance with the protocol approved by the Institutional Animal Care and Use Committee of Charles River Accelerator & Development Laboratories (Shanghai) Co., Ltd., Shanghai, China (Approval No. P202304060001).

Figure 16b displays in vivo recorded signals from all 12 channels. The lines beneath the waveforms mark spike events detected in real time by the detection algorithm embedded in the FPGA. Figure 16c,d showcase examples of individual neuron spikes extracted during a typical recording session. These results demonstrate that the system can accurately capture faint neural signals while efficiently performing FPGA-based data compression and wireless transmission, validating its potential for long-term multi-region brain activity monitoring.

## 5. Conclusions and Discussion

This paper presents the design and implementation of a wireless neural recording prototype system for multi-region brain activity monitoring. A parallel-link architecture with a shared central hub is proposed to address the limitations of conventional approaches. Compared with single-site wireless recorders, the ability to capture signals from multiple units in the same or different brain regions provides a clear advantage in observing diverse and comprehensive neuronal firing patterns. The core enabler of this prototype is the custom-designed 12-channel neural recording ASIC. The developed ASIC achieves a low IRN of 5.6 μVrms across the entire signal chain and occupies a silicon area of 4.84 mm × 0.36 mm. The ASIC integrates Manchester coding and an LVDS driver stage on-chip, supporting straightforward scaling toward high-channel-count applications without redesigning the analog front end. In vivo spike recordings in mice validate the efficacy of the proposed IC and system for neural spike acquisition.

Several limitations should also be noted. First, although the proposed architecture is intended for scalable multi-region brain activity monitoring, the present in -vivo experiments were performed in the CPu and did not demonstrate simultaneous recordings from multiple brain regions. Simultaneous multi-region recording will be further evaluated in future work. In addition, the animal experiments were conducted using three mice, with four repeated recordings over one month, which validates the repeated in -vivo recording capability of the proposed ASIC and recording module in a single brain region. However, these results should be regarded as preliminary repeated-recording evidence rather than a complete long-term stability validation. More systematic daily or weekly stability analysis, including quantitative evaluation of signal amplitude, noise level, channel yield, and spike waveform stability, will be conducted in future work.

Second, the measured total ASIC power consumption is approximately 10.13 mW under a 1.8 V supply. This power is dissipated on a compact ASIC area of 4.84 mm × 0.36 mm, and therefore its potential thermal effect should be carefully considered. Previous studies have commonly used a local tissue temperature rise of approximately 1 °C as a conservative thermal-safety limit for implantable neural devices. Numerical and experimental studies have shown that the allowable power dissipation strongly depends on chip size, heat-source distribution, packaging structure, implantation location, and heat-spreading path [28,29,30]. For example, under a 1 °C temperature-rise constraint, the maximum allowable power for compact cortical implant chips may be only several milliwatts, whereas a higher power budget may be tolerated when the heat source is distributed or when a more favorable packaging configuration is used. Thus, the thermal effect of dissipating 10.13 mW on the compact ASIC area in this prototype should be further evaluated through thermal simulation and direct tissue temperature-rise measurement.

In this prototype, the dominant power contributor is the standard LVDS driver, which draws approximately 5.3 mA consuming 9.55 mW. In contrast, the 12 recording channels consume only 0.58 mW in total, corresponding to 48.5 μW/channel. The standard LVDS interface was adopted to ensure robust differential transmission, common-mode noise rejection, and interference tolerance during in vivo validation. However, it is not power-optimal for long-term implantable applications. Future designs will replace the standard LVDS driver with a lower-power data-transmission circuit and will further reduce the transmitted data rate through event-driven telemetry and on-module data compression. For example, event-driven telemetry, spatiotemporal-correlation-based compression, and sparsity-aware data reduction have been shown to effectively reduce data rate and dynamic transmission power in high-channel-count neural recording systems [31,32,33]. In addition, thermal simulation, direct tissue temperature-rise measurement, battery-lifetime evaluation, and long-term recording feasibility will be systematically investigated in future work.

Third, the parallel-link architecture improves module-level fault isolation and avoids cascading failures that may occur in shared serial or daisy-chain topologies. However, each remote module requires an independent four-wire link to the central hub; therefore, the total wire count increases with the number of modules. This may increase the mechanical burden on freely moving animals and limit the scalability of the system for very high-density multi-region recordings. Future systems may improve scalability by increasing the channel count of each remote ASIC, using lightweight flexible interconnects, sharing supply lines among nearby modules, and adopting a hybrid hierarchical architecture in which local modules are first aggregated before being connected to the central hub.

Finally, this work mainly focuses on ASIC and system-level validation. Systematic bending/stretching tests, long-term wet-environment reliability tests, cytotoxicity tests, and chronic biocompatibility evaluations of the probe-interconnect assembly were not included in this study. These evaluations will be conducted in future work to further assess the mechanical reliability, encapsulation stability, and biocompatibility of the system for long-term implantation.

Table 1 summarizes the performance of the proposed system and provides a comprehensive comparison with existing state-of-the-art technologies.

## Figures and Tables

**Table 1:** Performance summary and comparison.

Parameter	[6]	[11]	[16]	This Work
Chip Technology (nm)	RHD2164	65	180	180
Supply (V)	3.4	0.6–1	1.8	1.8
Power (mW)	1480 *	<0.03	-	10.13
Data Communication I/O (No.)	SPI (10)	RF (-)	NeuroBus (7)	Serial (4)
Volume (mm^3^)	352 **	0.01–0.1	-	14
No. Electrodes	64	1	N × 30 ***	N × 12 ***
Electrode type	Passive	Passive	Passive	Passive
Recording	LFP/AP	ECoG	ECoG	LFP/AP
Area (mm^2^)	7.3 × 4.2	0.42	0.1	5 × 0.3
Power/Ch (μW)	526.71	<0.03	46.9	48.5
Gain (dB)	45.67	-	-	62.9
IRN (μVrms)	2.4	2.2 (500 Hz)	5.35 (0.3–7.5 kHz)	5.6 (0.25–10 kHz)
Resolution (bit)	16/-	8/-	-/9.92	12/10
Sampling (kSps)	30	0.5	15	28.93

* Includes 16 modules, FPGA, and other peripheral circuitry; ** Assuming a standard PCB thickness of 1.6 mm; *** N = number of parallelized daisy chains.

## Data Availability

Data is contained within the article.

## Abbreviations

The following abbreviations are used in this manuscript:

M1	Primary motor cortex
PMd	Dorsal premotor cortex
PMv	Ventral premotor cortex
PFC	Prefrontal cortex
ASIC	Application-Specific Integrated Circuit
LVDS	Low-Voltage Differential Signaling
MDP	Modular digital pixel
OSC	Oscillator
GDC	Global Digital Controller
AFE	Analog Front-End
SAR	Successive approximation register
P-S	Parallel-to-Serial
PMU	Power Management Unit
BLE	Bluetooth Low-Energy
LNA	Low-noise amplifier
BPF	Band-pass filter
BUFF	Buffer
BCG	Bias current generator
OTA	Operational Transconductance Amplifier
LPF	Local Field Potential
AP	Action Potential
EOC	End-of-Conversion
SI	Serial Input
SO	Serial Output
DFF	D-flip-flop
PTAT	Proportional to Absolute Temperature
CTAT	Complementary to Absolute Temperature
FSM	Finite State Machine
IRN	Input-referred noise
SNDR	Signal-to-noise and distortion ratio
ENOB	Effective number of bits
CPu	Caudate Putamen
